# Preparation and Enhanced Oil Recovery Mechanisms of Janus-SiO_2_-Reinforced Polymer Gel Microspheres

**DOI:** 10.3390/gels11070506

**Published:** 2025-06-30

**Authors:** Fei Gao, Baolei Liu, Yuelong Liu, Lei Xing, Yan Zhang

**Affiliations:** 1 National Engineering Research Center for Oil & Gas Drilling and Completion Technology, School of Petroleum Engineering, Yangtze University, Wuhan 430100, China; 501092@yangtzeu.edu.cn (F.G.); yanzhang@yangtzeu.edu.cn (Y.Z.); 2 Hubei Key Laboratory of Oil and Gas Drilling and Production Engineering (Yangtze University), Wuhan 430100, China; 3 State Key Laboratory of Low Carbon Catalysis and Carbon Dioxide Utilization (Yangtze University), Wuhan 430100, China; 4 Sinopec North China Company, Zhengzhou 450000, China; liuylong.hbsj@sinopec.com; 5 PetroChina Changqing Oilfield Shale Oil Development Branch, Qingyang 745100, China; xingleipetrochina@163.com

**Keywords:** SiO_2_ nanoparticle, amphiphilicity, polymer gel microspheres, conformance control, enhanced oil recovery

## Abstract

In order to improve oil recovery efficiency in low-permeability reservoirs, this study developed amphiphilic Janus-SiO_2_ nanoparticles to prepare polymer gel microspheres for enhanced oil recovery (EOR). Firstly, Janus-SiO_2_ nanoparticles were synthesized via surface modification using (3-aminopropyl)triethoxysilane and α-bromoisobutyryl bromide. Fourier-transform infrared spectroscopy (FTIR) and scanning electron microscopy (SEM) characterization confirmed the successful grafting of amino and styrene chains, with the particle size increasing from 23.8 nm to 32.9 nm while maintaining good dispersion stability. The Janus nanoparticles exhibited high interfacial activity, reducing the oil–water interfacial tension to 0.095 mN/m and converting the rock surface wettability from oil-wet (15.4°) to strongly water-wet (120.6°), thereby significantly enhancing the oil stripping efficiency. Then, polymer gel microspheres were prepared by reversed-phase emulsion polymerization using Janus-SiO_2_ nanoparticles as emulsifiers. When the concentration range of nanoparticles was 0.1–0.5 wt%, the particle size range of polymer gel microspheres was 316.4–562.7 nm. Polymer gel microspheres prepared with a high concentration of Janus-SiO_2_ nanoparticles can ensure the moderate swelling capacity of the particles under high-temperature and high-salinity conditions. At the same time, it can also improve the mechanical strength and shear resistance of the microspheres. Core displacement experiments confirmed the dual synergistic effect of this system. Polymer gel microspheres can effectively plug high-permeability zones and improve sweep volume, while Janus-SiO_2_ nanoparticles enhance oil displacement efficiency. Ultimately, this system achieved an incremental oil recovery of 19.72%, exceeding that of conventional polymer microsphere systems by more than 5.96%. The proposed method provides a promising strategy for improving oil recovery in low-permeability heterogeneous reservoir development.

## 1. Introduction

Crude oil remains a cornerstone of the global energy supply and serves as an essential raw material for the chemical industry, playing an important role in modern industrial development [1,2]. With the continuous exploitation of conventional oil and gas resources, the global petroleum industry has gradually shifted its focus toward the exploitation of unconventional resources, particularly low-permeability reservoirs [3,4]. However, low-permeability reservoirs are typically characterized by complex pore structures, narrow pore throats, and strong heterogeneity, which pose significant challenges to traditional water flooding techniques [5,6,7]. The main challenge is the severe reservoir heterogeneity, which often leads to water breakthrough along high-permeability zones, thereby bypassing substantial volumes of oil in low-permeability zones. Moreover, the narrow pore-throat architecture significantly increases capillary resistance, severely limiting the water flooding displacement efficiency even within zones already swept by injected water. As a result, both sweep efficiency and displacement efficiency remain low, and the average oil recovery of low-permeability reservoirs is often below 30% [8]. Therefore, developing novel technologies capable of simultaneously improving both sweep volume and displacement efficiency has become a critical scientific and technical challenge in enhancing the oil recovery of low-permeability reservoirs.

Recent research efforts have focused on the design of advanced functional materials that can achieve both in-depth fluid diversion and interfacial property optimization [9,10,11]. Among these, polymer gel microspheres have demonstrated significant potential for conformance control due to their controllable particle sizes (ranging from nanometers to micrometers) and swelling characteristics upon water adsorption [12,13,14]. These microspheres can efficiently plug high-permeability channels by in situ expansion, improving sweep efficiency, while their particle-size matching capacity allows for deep reservoir entrance [15,16,17,18,19,20]. Typically, such microspheres are synthesized using inverse emulsion polymerization within emulsion systems composed of aqueous monomer solutions and oil phases [21,22]. However, conventional polymer gel microspheres face two major technical limitations: insufficient mechanical strength leading to particle degradation under shear forces encountered in the reservoir environment [23,24,25], and a lack of interface activity, resulting in a limited reduction in oil–water interfacial tension and consequently poor displacement efficiency [26,27,28].

To overcome these limitations, amphiphilic Janus nanoparticles with asymmetric architectures have received a lot of attention due to their unique interfacial behavior and physicochemical properties [24,29,30,31]. Studies have shown that Janus nanoparticles not only exhibit surfactant-like amphiphilic properties that can substantially reduce oil–water interfacial tension and improve displacement efficiency but also exhibit excellent emulsifying capabilities as emulsifiers [32,33,34]. For example, Wu et al. [34] demonstrated that Janus-SiO_2_ nanoparticles could alter the rock wettability from oil-wet to water-wet and reduce the oil–water interfacial tension from 30 mN/m to 2.28 mN/m at a concentration of just 0.05 wt%. Furthermore, core flooding experiments conducted under reservoir conditions (50 °C, 50,000 mg/L salinity) revealed that the injection of 4 PV (pore volume) of the 0.01 wt% Janus-SiO_2_ nanoparticle dispersion following water flooding resulted in an additional oil recovery of 15.74%. These findings suggest that integrating amphiphilic Janus-SiO_2_ nanoparticles with polymer gel microspheres to form composite systems may provide a promising approach that couples in-depth fluid diversion capability with efficient oil displacement performance, thereby offering a novel solution for enhancing oil recovery in low-permeability reservoirs [35,36].

Among the available synthesis methods, the template technique remains the primary strategy for preparing Janus nanoparticles with controlled anisotropy, generally classified into two main approaches: hard-templating and soft-templating [37,38,39]. The hard-templating method typically consists of three steps: placing target nanoparticles on template surfaces, selective chemical alteration of exposed hemispheres, and template removal by physical or chemical means [40,41]. Although this approach enables precise structural control and accommodates diverse material types, it is constrained by complex processing steps and low production efficiency. In contrast, soft-templating techniques represented by Pickering emulsion methods have attracted interest due to their simplified preparation procedures (ambient temperature/pressure operation) and mild reaction conditions (avoiding strong acid/base treatments). This approach utilizes liquid–liquid interfaces as reaction platforms, achieving the directional assembly and asymmetric functionalization of nanoparticles by regulating interfacial tension and phase composition [42,43]. Yin et al. [44] first synthesized organic–inorganic hybrid Janus-SiO_2_ nanoparticles through the Pickering emulsion method. These nanoparticles were subsequently used as emulsifiers to stabilize styrene monomers. Following the addition of polymerization initiators, the Janus nanoparticles (PS-SiO_2_) were prepared. Tang et al. [32] used a wax-in-water Pickering emulsion system, controlling the directional arrangement of SiO_2_ nanoparticles at the oil–water interface to selectively shield one side with the wax phase, thereby achieving amino functionalization on the other side, ultimately obtaining amphiphilic Janus-SiO_2_ nanoparticles. The current design methods for enhancing oil recovery using composite systems simply combine surfactants with polymer gel particles, which have two key limitations: (1) surfactant performance degrades under high-salinity conditions due to reduced interfacial activity and significant adsorption losses, and (2) surfactants cannot structurally reinforce gel particles. Our Janus-SiO_2_ nanoparticles have both interfacial modifiers and structural reinforcers. The amphiphilic Janus-SiO_2_ can provide excellent interfacial activity that remains effective even in high-salinity environments, while their physical incorporation into the polymer matrix enhances mechanical strength and thermal stability. Because of the distinctive advantages of Janus-SiO_2_ nanoparticles in enhancing oil displacement efficiency and improving polymer microsphere performance, this study conducted a comprehensive investigation. Firstly, amphiphilic Janus-SiO_2_ nanoparticles were prepared through the soft-template method and characterized by Fourier-transform infrared spectroscopy (FTIR) and scanning electron microscopy (SEM) to analyze their chemical composition and morphological features. Their interfacial activity and ability to modify rock wettability were also evaluated. Subsequently, using these Janus-SiO_2_ nanoparticles as emulsifiers and acrylamide/acrylic acid as comonomers, a polymer microsphere system was constructed through emulsion polymerization, whose swelling properties were investigated. Finally, core displacement experiments were conducted to evaluate their enhanced oil recovery performance under low-permeability reservoir conditions, providing a novel technical approach for the efficient development of low-permeability reservoirs.

## 2. Results and Discussion

### 2.1. Characterization of Janus-SiO_2_ Nanoparticles

#### 2.1.1. Chemical Structure Analysis

**Figure 1:**
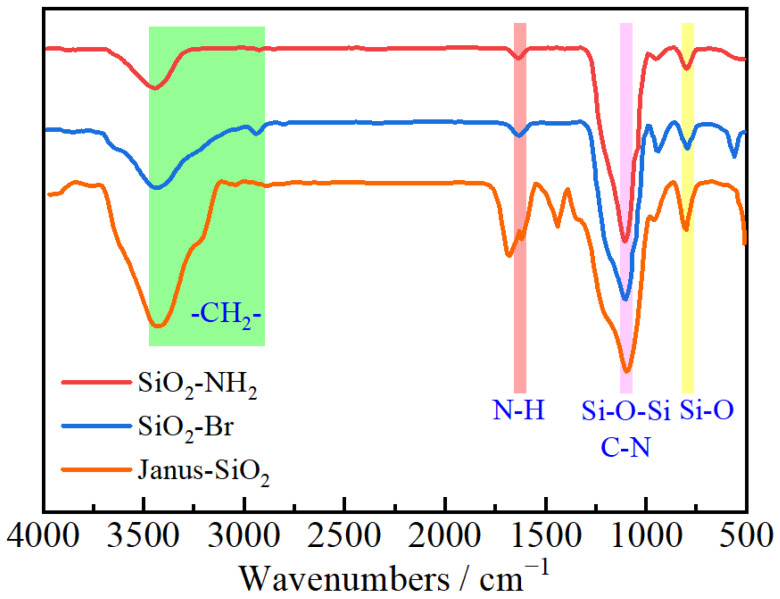
Infrared spectrogram of Janus-SiO_2_ nanoparticles.

Figure 1 presents the FTIR spectra in different stages of Janus-SiO_2_ nanoparticle synthesis. In the amination modification stage (SiO_2_-NH_2_), the characteristic absorption peaks at 1109 cm^−1^ and 804 cm^−1^ correspond to the stretching vibrations of Si-O-Si and Si-O bonds, respectively, with the peak at 1109 cm^−1^ also containing contributions from the C-N stretching vibration of silane coupling agent KH550. The value of 1640 cm^−1^ is the absorption peak of the bending vibration of the N-H bond. All these indicate the successful amination modification of SiO_2_ nanoparticles [24]. The spectrum of the bromination modification stage (SiO_2_-Br) shows a new characteristic peak at 560 cm^−1^ attributed to C-Br stretching vibration, providing direct evidence for the bromination modification of SiO_2_-NH_2_ nanoparticles. In the final product spectrum (Janus-SiO_2_), characteristic peaks of amide I and amide II bands appear at 1689 cm^−1^ and 1617 cm^−1^ respectively, accompanied by a new peak at 1440 cm^−1^ corresponding to benzene ring vibration [31]. The appearance of these characteristic peaks demonstrates the successful preparation of Janus-SiO_2_ nanoparticles using acrylamide and styrene as monomers.

#### 2.1.2. Particle Size Distribution and Microscopic Morphology

**Figure 2:**
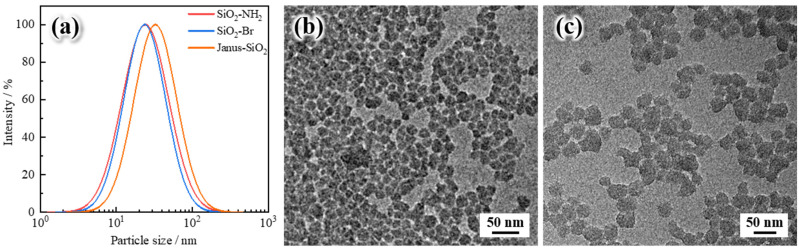
Particle size distribution and microscopic morphology: (**a**) particle size distribution of different SiO_2_ nanoparticles, (**b**) SEM image of the original SiO_2_ nanoparticles, and (**c**) SEM image of the Janus-SiO_2_ nanoparticles.

As shown in Figure 2a, the dynamic light scattering (DLS) analysis reveals significant changes in the size distribution of SiO_2_ nanoparticles before and after Janus modification. The original SiO_2_ nanoparticles exhibited a narrow size distribution, with an average particle size of approximately 23.8 nm, indicating good dispersion and uniformity. After Janus functionalization, the average particle size increased to 32.9 nm, representing an approximately 38.1% increase. This size growth is primarily attributed to the successful surface grafting of organic functional groups during the stepwise amination, bromination, and polymerization processes [28]. The formation of asymmetric polymer layers on the nanoparticle surface alters the solvation shell, resulting in a larger hydrodynamic radius detected by DLS [30]. Figure 2b,c present the scanning electron microscopy (SEM) images of the nanoparticles before and after Janus modification, respectively. The particle sizes estimated from SEM images are in good agreement with the DLS measurements, further validating the uniform growth in particle size upon functionalization. The morphological integrity of the nanoparticles was retained throughout the modification process [36].

#### 2.1.3. Interface Activity and Wettability

**Figure 3:**
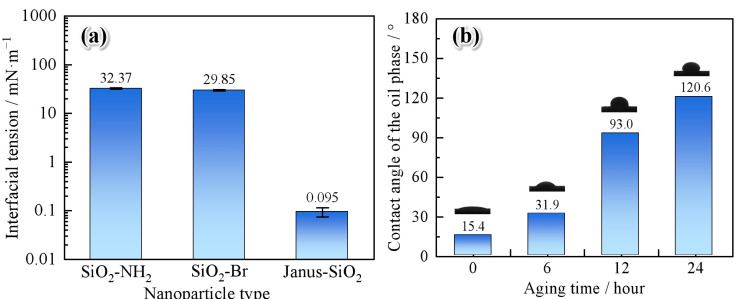
The ability to reduce the interfacial tension between oil and water and adjust rock wettability: (**a**) the effect of different SiO_2_ nanoparticles on reducing the oil–water interfacial tension; (**b**) the contact angle and photos of the rock surface after being immersed in Janus-SiO_2_ nanoparticles for different periods.

The Janus-SiO_2_ nanoparticles can significantly enhance oil displacement efficiency, and their ability to reduce the interfacial tension between oil and water is shown in Figure 3a. The experimental results proved that aminated and brominated SiO_2_ nanoparticles (0.3 wt%) can reduce oil–water interfacial tension (IFT) to 29.85–32.37 mN/m, while amphiphilic Janus-SiO_2_ nanoparticles exhibit higher interfacial activity, further lowering the tension to 0.095 mN/m (on the order of 10^−2^ mN/m). This low IFT value is comparable to that of commonly used surfactants such as petroleum sulfonates and betaines, indicating the strong interfacial activity of the Janus structure [45]. The decrease in interfacial tension is due to the amphiphilic structure of Janus-SiO_2_ nanoparticles. When Janus-SiO_2_ nanoparticles adsorb at the oil–water interface, the nanoparticles orient themselves with their hydrophilic amino-modified hemisphere extending into the aqueous phase and hydrophobic polystyrene-grafted hemisphere penetrating the oil phase. This special orientation formed by the interface monolayer can significantly reduce the oil–water interfacial tension. The reduction in IFT leads to a significant increase in the capillary number, thereby lowering capillary forces and promoting the stripping of residual oil [46]. Such behavior is especially beneficial for oil displacement in low-permeability or oil-wet reservoirs where capillary resistance plays a dominant role.

To further investigate the wettability alteration mechanism, we investigated the effect of Janus-SiO_2_ nanoparticle treatment on core surface wettability (Figure 3b). The untreated core surface showed an oil contact angle of 15.4°, confirming typical oil-wet characteristics. After 0.1 wt% Janus-SiO_2_ nanoparticle treatment, the contact angle increased steadily with treatment duration and reached 120.6° after 24 h, indicating a complete transition from oil-wet to water-wet. This significant wettability reversal is caused by the unique dual-affinity architecture of Janus particles [33]. Upon adsorption onto the rock surface, the hydrophobic side of the nanoparticles (e.g., grafted polystyrene chains) anchors firmly to the oil-wet surface through van der Waals and hydrophobic interactions, while the hydrophilic side (e.g., amino-functionalized surface) remains exposed to the aqueous phase. This directional orientation reconstructs a stable hydrophilic interface, facilitating spontaneous water imbibition into oil-filled pores [14]. Moreover, this wettability alteration mechanism inhibits the re-adsorption and regeneration of oil films on pore surfaces during subsequent flooding stages. Consequently, the synergistic effect of low-IFT and -wettability alteration significantly improves the oil displacement efficiency of the Janus-SiO_2_ nanoparticles under reservoir-relevant conditions.

### 2.2. Performances of Polymer Gel Microspheres

#### 2.2.1. Particle Size Distribution and Microscopic Morphology

**Figure 4:**
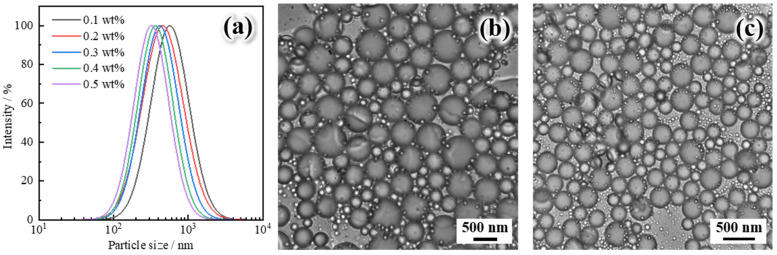
Particle size distribution and microscopic morphology: (**a**) particle size distribution of polymer gel microspheres reinforced with different Janus-SiO_2_ nanoparticles, (**b**) SEM image of polymer gel microspheres with 0.1 wt% Janus-SiO_2_ nanoparticles, and (**c**) SEM image of polymer gel microspheres with 0.5 wt% Janus-SiO_2_ nanoparticles.

Figure 4a presents the particle size distribution characteristics of polymer gel microspheres reinforced by Janus-SiO_2_ nanoparticles at different concentrations. It demonstrates an inverse correlation between the average particle size of microspheres and nanoparticle concentration. As the concentration of Janus-SiO_2_ nanoparticles increases from 0.1 wt% to 0.5 wt%, the average microsphere diameter decreases significantly from 562.7 nm to 316.4 nm. The DLS curves for all samples exhibit monomodal distribution profiles, indicating a high degree of size uniformity. This behavior can be attributed to the increased surface coverage and emulsification efficiency of Janus nanoparticles at higher concentrations, which leads to the formation of more stable and finer Pickering emulsions [36]. As is shown in Figure 4b,c, the SEM images confirm these findings, revealing uniformly spherical microspheres with no apparent aggregation or morphological irregularities. The effective size regulation achieved by controlling nanoparticle concentration provides a flexible approach for designing microspheres with desired dimensions to meet different reservoir conditions [37].

#### 2.2.2. Swelling Behavior

The swelling behavior of polymer gel microspheres reinforced by Janus-SiO_2_ nanoparticles in reservoir environments plays an important role in determining their performance in conformance control applications. Swelling allows microspheres to grow and selectively block high-permeability channels, which improves sweep efficiency. However, severe swelling might compromise mechanical strength. Therefore, a balanced swelling behavior is desired. The swelling capacity is closely related to temperature, salinity, and swelling duration.

**Figure 5:**
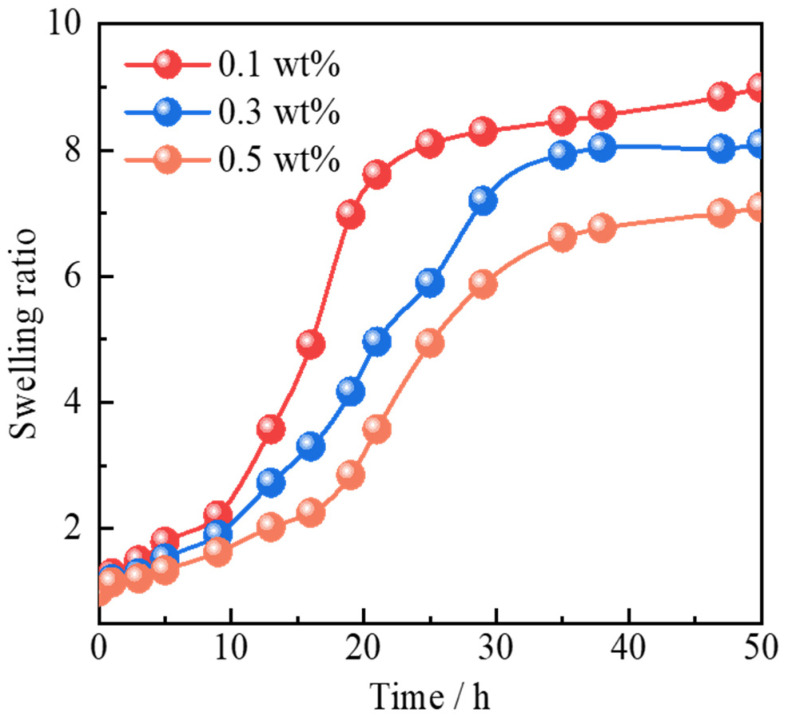
Swelling behavior of polymer gel microspheres reinforced by Janus-SiO_2_ nanoparticles.

As shown in Figure 5, the swelling kinetics of microspheres reinforced with different concentrations of Janus-SiO_2_ nanoparticles were investigated in formation water. As the nanoparticle concentration increases from 0.1 wt% to 0.5 wt%, the stable swelling ratio of the polymer gel microspheres gradually decreases from 8.10 to 6.77. This trend indicates that the presence of nanoparticles significantly inhibits the swelling behavior of polymer gel microspheres [23]. More importantly, the time required to reach swelling equilibrium is also significantly prolonged with increasing nanoparticle concentration, extending from 25 h to 38 h. This dual effect can be attributed to the special distribution state of Janus-SiO_2_ nanoparticles on the surface of polymer gel microspheres [13]. Their amphiphilic structure enables them to adsorb and form a semi-permeable shielding layer on the microsphere surface, which reduces the effective contact area between the hydrophilic polymer matrix and surrounding water molecules. Additionally, the nanoparticle layer can delay water penetration, thereby appropriately inhibiting the rapid water absorption and swelling of the microspheres [16]. Such appropriate swelling not only prevents over-expansion but also improves the structural strength of microspheres under reservoir conditions, effectively overcoming the common drawback of strength decrease in traditional polymer gel microspheres.

**Figure 6:**
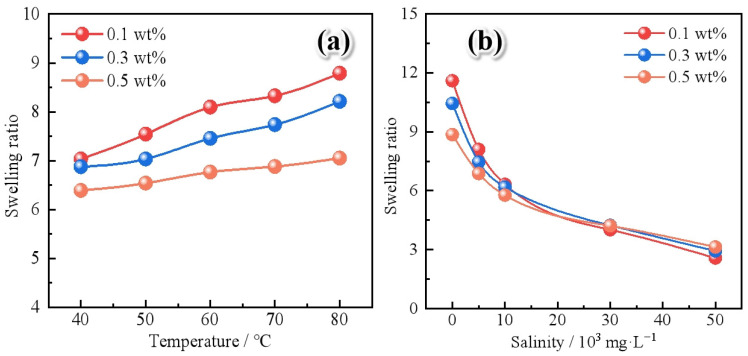
The effect of reservoir conditions on the swelling behavior of the polymer gel microspheres reinforced by Janus-SiO_2_ nanoparticles: (**a**) temperature and (**b**) salinity of the formation water.

The effect of temperature on the swelling behavior of polymer gel microspheres is shown in Figure 6a. In the temperature range of 40 °C to 80 °C, the final swelling ratio of the microspheres significantly increases with the increase in temperature. This is because high temperature enhances the interaction between water molecules and hydrophilic groups such as amide groups in the polymer network, promoting the formation of more stable solvation layers. However, this temperature-induced swelling can also reduce the injectivity of microspheres during the injection process, as premature expansion may hinder their migration through pore throats [29]. The temperature-induced swelling enhancement becomes less significant as the nanoparticle concentration increases. This is because the interfacial confinement imposed by Janus-SiO_2_ nanoparticles restricts the expansion space of the polymer microsphere. Figure 6b presents the effect of salinity on microsphere swelling performance. At a fixed nanoparticle content of 0.3 wt%, the swelling ratio decreases significantly from 10.44 to 2.93 as the salinity of formation water increases from 0 to 50,000 mg/L. The presence of electrolytes compresses the electrical double layer and reduces the osmotic driving force for water adsorption, leading to a denser polymer network and lower swelling capacity. When the nanoparticle concentration increased from 0.1 wt% to 0.5 wt%, the negative effect of salinity on swelling was reduced [31]. The physical barrier formed by Janus-SiO_2_ nanoparticles restricts the penetration of electrolyte ions into the microsphere core, thereby reducing the ionic shielding effect on charged groups within the polymer. This confers enhanced salt resistance to the microspheres, enabling them to maintain more stable swelling behavior in high-salinity environments. The presence of Janus-SiO_2_ nanoparticles alleviated the influence of formation temperature and salinity, regulated the expansion kinetics, and enhanced the mechanical stability under harsh reservoir conditions [35].

#### 2.2.3. Shearing Resistance

The shearing stability of polymer gel microspheres reinforced by amphiphilic Janus-SiO_2_ nanoparticles was evaluated by high-speed shearing tests to simulate mechanical stress conditions encountered during injection and migration in porous media. The experiments were conducted using a Waring Laboratory Blender equipped with a stainless-steel shear head. The shear parameter was set to 12,000 rpm [25]. Microspheres containing different concentrations of Janus-SiO_2_ nanoparticles were tested to assess the influence of nanoparticle content on particle size retention under mechanical stress [29].

**Figure 7:**
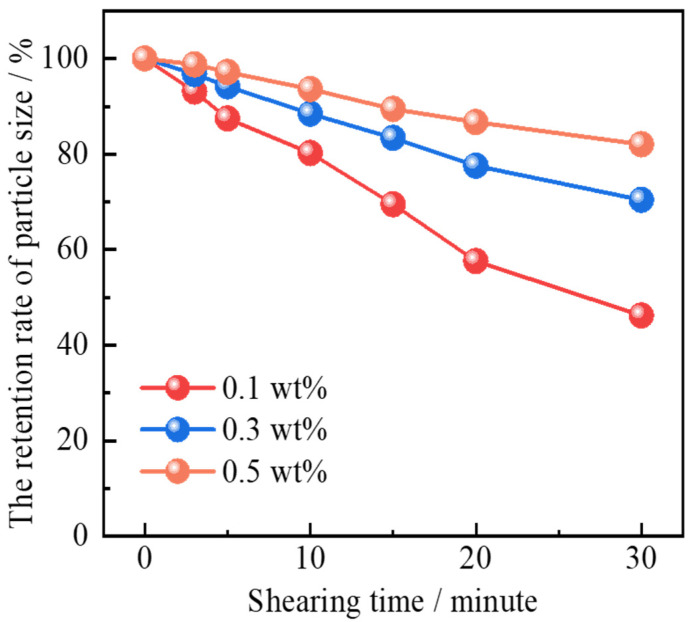
Shearing resistance of polymer gel microspheres reinforced by Janus-SiO_2_ nanoparticles.

As is shown in Figure 7, a clear positive correlation is observed between nanoparticle concentration and shearing resistance. Specifically, as the nanoparticle concentration increased from 0.1 wt% to 0.5 wt%, the particle size retention rate after 30 min of shearing significantly improved. At 0.5 wt% concentration, the microspheres retained approximately 82.1% of their particle size, indicating excellent structural stability under high-shear conditions. This enhanced shearing stability is primarily attributed to the protective interfacial layer formed by the amphiphilic Janus-SiO_2_ nanoparticles adsorbed on the microsphere surface [45]. These nanoparticles create a physically reinforced shell that provides mechanical shielding against external forces.

### 2.3. Injection and Plugging Capacity

**Figure 8:**
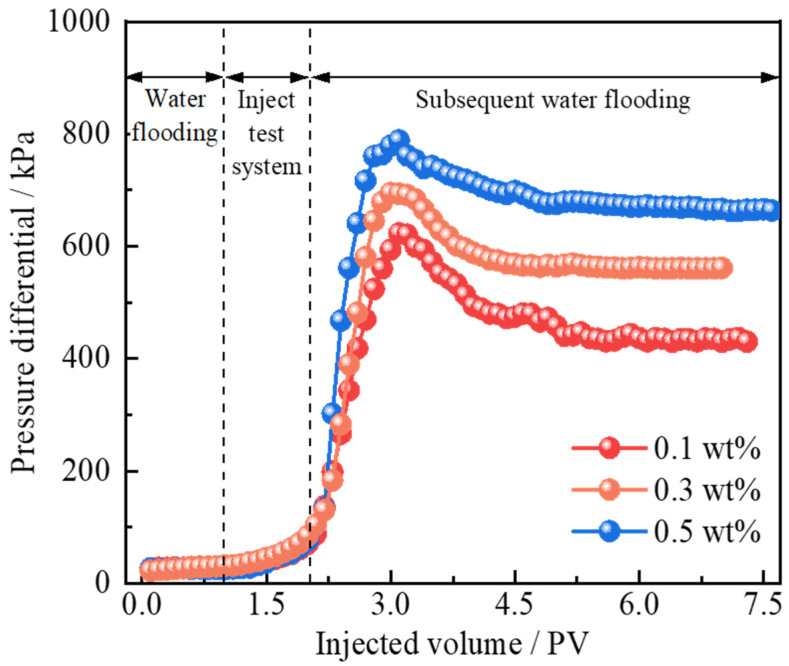
The displacement pressure of polymer gel microspheres reinforced by Janus-SiO_2_ nanoparticles of different concentrations varies with the injection volume.

The plugging performance of polymer gel microspheres reinforced by Janus-SiO_2_ nanoparticles in high-permeability channels is an important factor that directly influences the sweep efficiency of subsequent water flooding. In this study, core displacement experiments were conducted to evaluate the injection behavior and plugging capacity of polymer gel microspheres with varying concentrations (0.1, 0.3, and 0.5 wt%) of Janus-SiO_2_ nanoparticles. The pressure differential evolution and corresponding plugging rates are presented in Figure 8 and Table 1.

Figure 8 presents the displacement pressure characteristics with polymer gel microspheres reinforced by Janus-SiO_2_ nanoparticles at different concentrations. The experimental results demonstrate the smooth injection of all polymer gel microspheres, with the pressure differential showing a progressive increase with injected pore volumes. This process reflects the multiple mechanisms including direct pore passage, deformable migration through constrictions, mechanical trapping, and delayed swelling-induced plugging [30]. As the Janus-SiO_2_ nanoparticle concentration increased from 0.1 to 0.5 wt%, the reduction in average particle size resulted in a moderate decrease in initial injection pressure. This is attributed to the smaller and more uniform microsphere size at higher nanoparticle concentrations, facilitating smoother pore entry and transport. Following a 72 h aging period under reservoir conditions, subsequent water flooding caused a sharp rise in pressure differential, indicating the successful swelling and accumulation of microspheres within pore throats [31]. The peak breakthrough pressure increased significantly with nanoparticle concentration, and the microspheres with 0.5 wt% Janus-SiO_2_ showed a 26.7% higher breakthrough pressure than that of the 0.1 wt% group. This enhancement is because of the interfacial reinforcement effect imparted by Janus-SiO_2_ nanoparticles, which improve both the structural integrity and plugging capacity of the microspheres [33]. Although the pressure differential declined somewhat post breakthrough due to the formation of new preferential flow channels, the final stabilized pressure was still significantly higher than the initial state. This suggests that residual microspheres continued to exert plugging influence within the pore throats [38].

Table 1 further proves these findings by quantifying the plugging rate, which showed a clear positive correlation with nanoparticle content. When the concentration increased from 0.1 wt% to 0.5 wt%, the plugging rate increased from 93.08% to 96.32%. These results demonstrate the excellent conformance control capabilities of polymer gel microspheres reinforced by Janus-SiO_2_ nanoparticles in heterogeneous reservoirs. The amphiphilic nanoparticles not only help with emulsification and particle size control, but they can also improve the adaptability and plugging strength of the microspheres under complex reservoir conditions. Such improvements are essential for achieving efficient sweep redistribution and minimizing water channeling in enhanced oil recovery applications.

### 2.4. The Potential for Enhanced Oil Recovery (EOR)

To evaluate the performance of polymer gel microspheres reinforced by amphiphilic Janus-SiO_2_ nanoparticles for enhanced oil recovery, a comparative core displacement experiment was conducted using three systems: (1) 0.3 wt% amphiphilic Janus-SiO_2_ nanoparticles, (2) 0.5 wt% conventional polymer gel microspheres, and (3) 0.5 wt% polymer gel microspheres reinforced by 0.3 wt% amphiphilic Janus-SiO_2_ nanoparticles. A double-layer heterogeneous core model was used to simulate reservoir heterogeneity, beginning with water flooding until the water-cut exceeded 98%, followed by the injection of a 1.0 PV test system, aging at 70 °C for 3 days, and subsequent secondary water flooding, with the continuous monitoring of water-cut, oil recovery, and displacement pressure.

**Figure 9:**
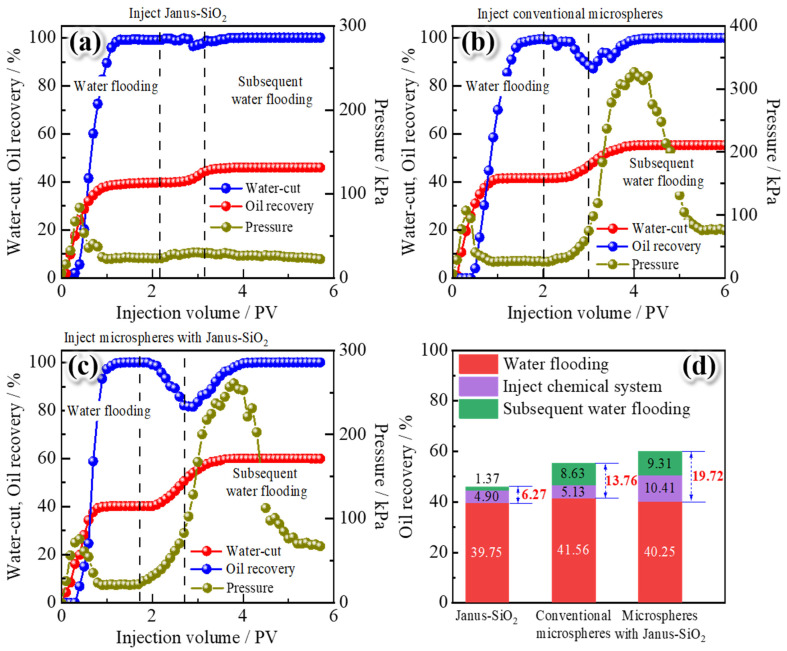
A comparison of enhanced oil recovery properties of different test systems: (**a**) amphiphilic Janus-SiO_2_ nanoparticles, (**b**) conventional polymer gel microspheres, (**c**) polymer gel microspheres reinforced with Janus-SiO_2_ nanoparticles, and (**d**) oil recoveries in different stages of the cores treated with different systems.

As shown in Figure 9a, the single Janus-SiO_2_ nanoparticle system achieved an incremental oil recovery of 6.27%. Although this system effectively reduced the oil–water interfacial tension and altered the rock surface wettability, its impact on sweep efficiency was limited. The pressure curve indicated that both the nanoparticles and the following water primarily flowed along pre-existing dominant channels formed during initial water flooding. The pressure curve showed that the injectability of Janus-SiO_2_ nanoparticles is very good, with a very small pressure increase. The nanoparticles and the subsequent injected water mainly flow along the pre-existing dominant channels formed during the initial water flooding [6]. As a result, oil displacement was restricted to the high-permeability zones and adjacent areas, causing a poor effect of enhanced oil recovery. The conventional polymer gel microspheres exhibited better conformance control performance with 13.76% incremental recovery. As shown in Figure 9b, the polymer gel microspheres can be smoothly injected into the core, with a limited increase in injection pressure. During this process, the microspheres can block the dominant channels in the high-permeability layer. Subsequently, the injected water is diverted to areas that have not been swept before, and the displacement pressure increases in the subsequent water flooding stage. Due to the improvement in sweep volume, a large amount of crude oil in the originally unswept areas has been displaced, thereby enhancing the oil recovery [11], while conventional polymer gel microspheres lack interfacial activity, limiting their ability to displace residual oil in swept zones. The polymer gel microspheres reinforced with Janus-SiO_2_ nanoparticles achieved an optimal incremental recovery of 19.72%. As illustrated in Figure 9c, the injection pressure of Janus SiO_2_-reinforced polymer gel microspheres was slightly higher than that of conventional polymer gel microspheres. This is because the rigid SiO_2_ nanoparticles inhibited the significant deformation of the microspheres, but the microspheres themselves were small, and the SiO_2_ particles slowed down the water absorption and expansion speed of the microspheres, so their injection performance was still good. In the subsequent water flooding process, due to the conformance control effect of the polymer gel microspheres, the injected water was diverted to areas that were not previously unswept [13]. Meanwhile, the stabilized flow redistribution enabled by microsphere plugging allowed the interfacial activity of Janus-SiO_2_ nanoparticles to continuously act on the remaining oil in the low-permeability zone. The amphiphilic Janus-SiO_2_ nanoparticles further improved the oil displacement efficiency by reducing interfacial tension, altering rock wettability, and facilitating the stripping of residual oil. The simultaneous impact of macroscopic conformance control and microscopic interfacial regulation resulted in a significant increase in swept volume and increased oil displacement efficiency. This dual action explains the reason why the system can achieve a 19.72% increase in recovery rate (5.96% higher than the conventional microspheres) [27].

### 2.5. The EOR Mechanism

**Figure 10:**
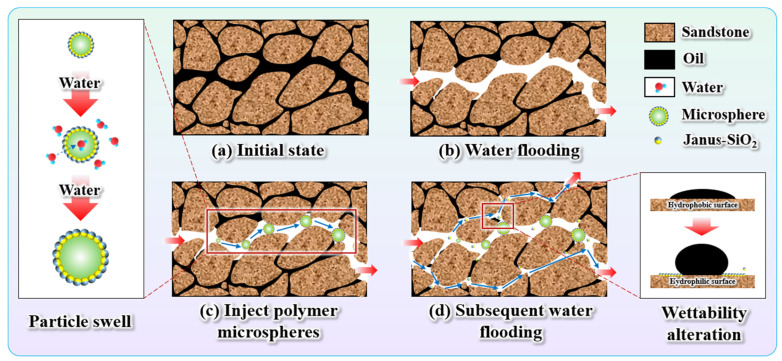
Schematic illustration of enhanced oil recovery mechanisms by the polymer gel microspheres reinforced with Janus-SiO_2_ nanoparticles.

Previous studies demonstrated that combining profile modification agents with interfacial modifiers could simultaneously improve sweep efficiency through in-depth profile control and enhance displacement efficiency via interfacial tension reduction [5,25,26,28], effectively solving the macroscopic sweep efficiency and microscopic displacement problems of highly heterogeneous reservoirs. Our findings extend this principle by introducing Janus nanoparticles as both emulsifiers and interfacial modifiers. Based on the results of the core displacement experiments, a synergistic enhanced oil recovery (EOR) mechanism for the polymer gel microspheres reinforced with Janus-SiO_2_ nanoparticles is proposed, as illustrated in Figure 10.

Figure 10a,b depict the typical water flooding processes of heterogeneous reservoirs. Injected water tends to preferentially flow through high-permeability zones, resulting in a water breakthrough and the formation of residual oil in swept areas. In contrast, low-permeability layers experience insufficient displacement pressure, leading to a high remaining oil saturation due to ineffective sweeping [14]. As shown in Figure 10c,d, after injecting the polymer gel microspheres reinforced with Janus-SiO_2_ nanoparticles, the system exhibited a dual synergistic mechanism involving both conformance control and interfacial regulation. Specifically, the polymer gel microspheres physically plug the dominant pore throats through a combination of the mechanical accumulation and swelling effect, thus preventing water channeling. Simultaneously, the amphiphilic Janus-SiO_2_ nanoparticles reduce oil–water interfacial tension and alter the rock surface wettability from oil-wet to water-wet, thereby enhancing the stripping of oil adhering to pore surfaces [17]. This system that combines microspheres and Janus-SiO_2_ nanoparticles improves both macroscopic sweep efficiency and microscopic oil displacement efficiency. Therefore, the polymer gel microspheres reinforced with Janus-SiO_2_ nanoparticles improve oil recovery and have a high potential for application in heterogeneous reservoir development [1]. Furthermore, the adjustability of microsphere particle size enables it to adapt to reservoirs with different permeability and has wide applicability in heterogeneous reservoirs.

## 3. Conclusions

This study successfully developed a novel EOR system based on polymer gel microspheres reinforced by amphiphilic Janus-SiO_2_ nanoparticles, providing an innovative solution for the efficient development of low-permeability heterogeneous reservoirs. The main conclusions are as follows:

1. Amphiphilic Janus-SiO_2_ nanoparticles with controlled asymmetric wettability were successfully synthesized via a Pickering emulsion template method. FTIR and SEM characterization confirmed the successful surface modification with amino and bromo groups, with the average particle size increasing from 23.8 nm to 32.9 nm while maintaining excellent dispersibility.
2. The Janus-SiO_2_ nanoparticles demonstrated outstanding interfacial activity, effectively reducing the oil–water interfacial tension to 0.095 mN/m and altering the rock surface wettability from oil-wet to strongly water-wet, thereby significantly enhancing oil stripping efficiency.
3. Polymer gel microspheres were prepared by reversed-phase emulsion polymerization using Janus-SiO_2_ nanoparticles as emulsifiers. When the concentration range of nanoparticles was 0.1–0.5 wt%, the particle size range of polymer gel microspheres was 316.4–562.7 nm.
4. Polymer gel microspheres reinforced by Janus-SiO_2_ nanoparticles exhibit swelling behavior in response to temperature and salinity. Polymer gel microspheres prepared with a high concentration of Janus-SiO_2_ nanoparticles can ensure the moderate swelling capacity of the particles under high-temperature and high-salinity conditions. At the same time, it can also improve the mechanical strength and shear resistance of the microspheres.
5. Core displacement experiments demonstrated the dual synergistic effects of the polymer gel microspheres reinforced by Janus-SiO_2_ nanoparticles. On one hand, microspheres can effectively plug high-permeability zones (the plugging rate of the system with 0.5 wt% nanoparticles can reach 96.32%) and improve sweep volume, while Janus-SiO_2_ nanoparticles can further improve oil displacement efficiency. These combined effects have increased the oil recovery by 19.72%, which is more than 5.96% higher than that of the conventional microsphere system.
6. The expansion behavior of polymer gel microspheres is still difficult to control. Determining how to optimize the initial particle size of microspheres according to reservoir physical parameters (such as temperature, salinity, pore throat characteristics, etc.) to achieve the balance between injectivity and expansion plugging capacity is the key direction of future research. In addition, field tests are needed to verify the long-term stability of the system in a dynamic reservoir environment.

## 4. Materials and Methods

### 4.1. Materials

The nano-SiO_2_ particles used in this study were supplied by Shandong Wanhua Tianhe New Materials Co., Ltd. (Dongying, China) The surface modifying agent (3-aminopropyl)triethoxysilane (KH550), α-bromoisobutyryl bromide (BIBB), triethylamine, N,N′-methylenebisacrylamide, and N,N,N′,N″,N″-pentamethyldiethylenetriamine were purchased from Shanghai Aladdin Biochemical Technology Co., Ltd. (Shanghai, China) The acrylamide (AM), acrylic acid (AA), ammonium persulfate (APS), sodium bisulfite (NaHSO_3_), and styrene (St) were obtained from Shanghai Macklin Biochemical Technology Co., Ltd. (Shanghai, China) Organic solvents such as toluene and anisole, as well as inorganic salts, were provided by Sinopharm Chemical Reagent Co., Ltd. (Shanghai, China) The crude oil samples were collected from Shengli Oilfield in China, with a density of 0.85 g/cm^3^ and a viscosity of 12.84 mPa·s at 25 °C. The simulated formation water had a salinity of 13180.1 mg/L, with the detailed ionic composition listed in Table 2. The core samples were natural outcrop sandstones with dimensions of 2.50 cm in diameter and 10.00 cm in length.

### 4.2. Preparation of Amphiphilic Janus-SiO_2_ Nanoparticles

**Figure 11:**
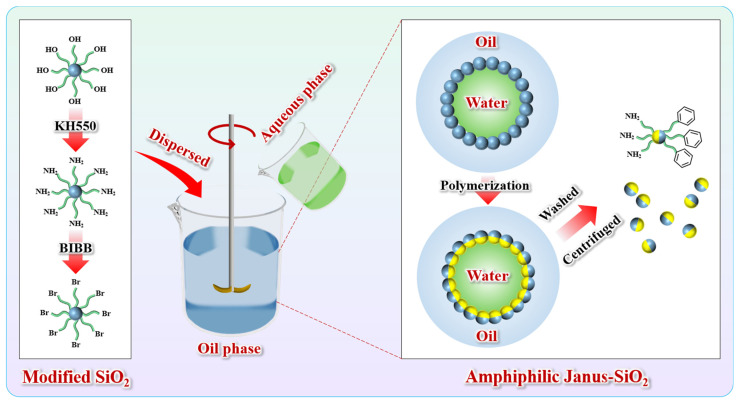
A schematic diagram of the preparation of amphiphilic Janus-SiO_2_ nanoparticles.

Amphiphilic Janus-SiO_2_ nanoparticles were synthesized through the Pickering emulsion method. Initially, the SiO_2_ nanoparticles were asymmetrically modified to exhibit distinct wettability on opposite sides. The amphiphilic architecture was subsequently stabilized through polymerization, and the final Janus-SiO_2_ nanoparticles were obtained after selective dissolution in organic solvents. The detailed synthesis process is illustrated in Figure 11.

#### 4.2.1. Amination of SiO_2_ Nanoparticles

A total of 1.5 g of SiO_2_ nanoparticles was dispersed in 50 mL of anhydrous ethanol and ultrasonicated for 30 min to ensure uniform dispersion. Then, 0.8 g of KH550 was slowly added, and the mixture was stirred at 70 °C and 200 rpm for 6 h. The resulting product was washed three times with anhydrous ethanol and vacuum-dried at 50 °C for 24 h to obtain aminated SiO_2_ nanoparticles (SiO_2_–NH_2_).

#### 4.2.2. Bromination of SiO_2_ Nanoparticles

Next, 0.1 g of SiO_2_-NH_2_ was dispersed in 50 mL of toluene and ultrasonicated for 30 min. Then, 2 mL of triethylamine was added under ice-water bath conditions, followed by the slow dropwise addition of 0.5 mL of BIBB. The reaction was maintained at 0 °C for 2 h and then allowed to proceed at room temperature for an additional 24 h. The final product was washed with toluene three times and vacuum-dried at 25 °C for 24 h to obtain brominated SiO_2_ nanoparticles (SiO_2_–Br).

#### 4.2.3. Preparation of Amphiphilic Janus-SiO_2_ Nanoparticles

For the Janus structure synthesis, 0.1 g of SiO_2_-Br was dispersed in 8 mL of anisole via ultrasonication. Then, 1.5 g of St was added to form the oil phase, while AM and N,N,N′,N″,N″-pentamethyldiethylenetriamine were dissolved in deionized water to form the aqueous phase. Both phases were purged with nitrogen for 30 min to remove dissolved oxygen. Under a nitrogen atmosphere and continuous stirring at 200 rpm, the aqueous phase was slowly introduced into the oil phase, followed by the addition of 30 mg of CuBr_2_ as a catalyst. The reaction was carried out at 70 °C for 6 h before termination. The product was sequentially washed with anhydrous ethanol and tetrahydrofuran three times, centrifuged, and vacuum-dried at 25 °C for 24 h to obtain the final amphiphilic Janus-SiO_2_ nanoparticles.

### 4.3. Preparation of Janus-SiO_2_-Reinforced Polymer Gel Microspheres

**Figure 12:**
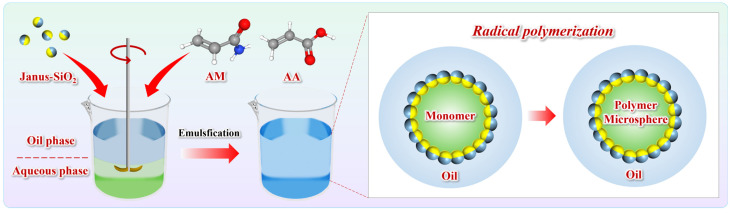
A schematic diagram of the preparation of polymer gel microspheres reinforced by amphiphilic Janus-SiO_2_ nanoparticles.

The polymer microsphere emulsion was prepared using industrial white oil as the oil phase and amphiphilic Janus-SiO_2_ nanoparticles as the Pickering emulsifier. The experimental procedure is illustrated in Figure 12. The aqueous phase was prepared by dissolving 40 wt% AM, AA, and 0.4 wt% MBA in deionized water. The mass ratio of the oil phase, emulsifier, and aqueous phase was set at 45:20:35. Before emulsification, both phases were purged with nitrogen for 30 min. The oil phase and emulsifier were first added to a three-neck flask and ultrasonicated for 30 min to ensure homogeneous dispersion. Under continuous stirring at 400 rpm, a small portion of the aqueous phase was introduced for pre-emulsification over 30 min. The pH of the system was adjusted to 7.0 ± 0.5 using NaOH solution. Then, under nitrogen protection, the remaining aqueous phase along with a co-initiator system of APS and NaHSO_3_ (0.5 wt% relative to total monomer content) was added dropwise at a constant rate. The reaction was then maintained at 45 °C for 4 h to obtain the polymer gel microspheres reinforced by amphiphilic Janus-SiO_2_ nanoparticles (JSPM).

### 4.4. Characterization of Janus-SiO_2_ Nanoparticles

#### 4.4.1. Fourier-Transform Infrared Spectroscopy (FTIR) Characterization

The chemical structure of synthesized Janus-SiO_2_ nanoparticles was characterized by FTIR spectroscopy using the KBr pellet method. Spectra were recorded in the range of 4000–400 cm^−1^ with 4 cm^−1^ resolution at 25 ± 1 °C.

#### 4.4.2. Scanning Electron Microscopy (SEM) Characterization

Dried samples were uniformly dispersed on conductive adhesive and sputter-coated with gold for 60 s to enhance conductivity. Morphological features were observed using SEM at 15 kV accelerating voltage.

#### 4.4.3. Dynamic Light Scattering (DLS) Analysis

The average particle diameters were measured by DLS at 25 °C. Samples were dispersed in deionized water and ultrasonicated for 30 min before measurement.

#### 4.4.4. Oil–Water Interfacial Tension Measurement

The interface tension test was used to verify the ability of Janus-SiO_2_ nanoparticles to reduce oil–water interface tension, which is the basis for enhancing oil displacement efficiency. Interfacial tension was determined using a spinning drop tensiometer at 70 °C. Specifically, 0.5 μL crude oil was injected into quartz capillaries filled with Janus-SiO_2_ dispersion. The interfacial tension was calculated using the morphological parameters of the stable oil droplets rotating at high speed.

#### 4.4.5. Wettability Measurement

The contact angle test was used to verify the ability of Janus-SiO_2_ nanoparticles to alter the wettability, which is the basis for enhancing oil displacement efficiency. Sandstone core slices were immersed in Janus-SiO_2_ dispersion for 24 h, and then vacuum-dried for 12 h. Static contact angles of oil droplets were measured at 25 °C.

### 4.5. Swelling Behavior Analysis

The analysis of swelling behavior is an important indicator for evaluating the potential of conformance control and the injectivity. The swelling behavior of polymer gel microspheres at different temperatures was investigated. An amount of 0.5 g of polymer gel microspheres was immersed in 100 mL of deionized water or saline solution and maintained at constant temperature for 72 h. The particle size distribution was periodically measured by DLS measurement to record the median diameter (*D*_50_) changes at different hydration times. The swelling ratio (*R*) was calculated according to Equation (1):

(1) $$R=\frac{D_{2}-D_{1}}{D_{1}}$$

where *D*_1_ and *D*_2_ represent the median diameters (μm) of polymer gel microspheres before and after swelling, respectively.

### 4.6. Conformance Control Experiment

**Figure 13:**
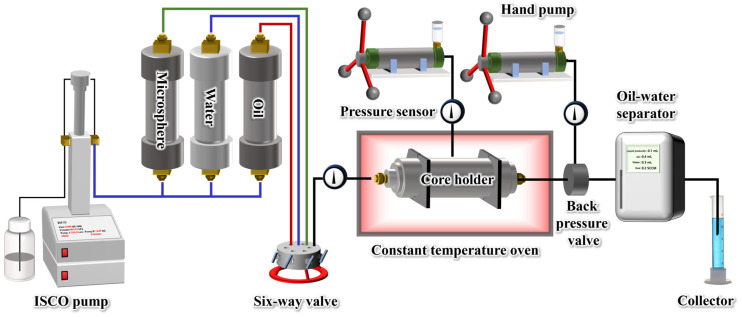
Schematic diagram of core displacement experimental device.

The conformance control performance of polymer gel microspheres was evaluated through core flooding experiments using the setup shown in Figure 13. The core was first ultrasonically cleaned with deionized water for 30 min and dried at 60 °C, and then its dimensions and porosity were measured. The core was then loaded into a core holder, and formation water was injected at a constant flow rate of 0.5 mL/min under 70 °C. After injected pressure stabilization, the initial permeability *k*_1_ was calculated by Darcy’s law. Subsequently, 1.0 PV (pore volume) of polymer microsphere solution (500 mg/L) was injected at the same flow rate. The core holder was sealed and maintained at 70 °C for 3 days. Formation water was then reinjected at 0.5 mL/min while the pressure was recorded in real-time. After pressure stabilization, the permeability *k*_2_ was calculated. During the experiment, the confining pressure was always 3.0 MPa higher than the injection pressure. The backpressure was set to 0.5 MPa. The plugging efficiency (*η*) was calculated using Equation (2).

(2) $$\eta =\frac{k_{1}-k_{2}}{k_{1}}$$

### 4.7. Oil Displacement Experiment

Based on the experimental setup shown in Figure 13, a double-layer heterogeneous core was used to evaluate the enhanced oil recovery performance of polymer gel microspheres. The double-layer heterogeneous core was self-made in the laboratory, with dimensions of 10 cm in length and 2.5 cm in diameter. The cleaned and dried core was saturated with crude oil through the vacuuming and pressurizing method and aged at 70 °C for 7 days. The prepared core was then loaded into the core holder with the confining pressure maintained at 3.0 MPa above the injected pressure and backpressure at 0.5 MPa. Formation water was first injected into the core and the injection was stopped when the water-cut exceeded 98% and stabilized. Then, 1.0 PV of the test system was injected at the same rate, followed by sealing the core holder for 3 days of aging at 70 °C. Finally, subsequent water flooding was carried out at 0.5 mL/min. Throughout the experimental process, the changes in injection pressure were continuously monitored and the oil/water content in the produced fluid was recorded.

## Figures and Tables

**Table 1:** Core parameters and plugging rate of core displacement experiment.

No.	Length/mm	Diameter/mm	Permeability/mD	Porosity/%	Nanoparticle Concentration/wt%	Plugging Rate/%
1	98.95	25.03	43.74	14.97	0.1	93.08
2	99.36	25.02	45.14	15.68	0.3	94.49
3	98.54	25.02	47.55	16.34	0.5	96.32

**Table 2:** Ion composition of simulated formation water.

Ions	Na^+^/K^+^	Mg^2+^	Ca^2+^	Cl^−^	SO_4_^2−^	CO_3_^2−^	HCO_3_^−^	Total
Concentration/mg·L^−1^	4392.0	39.2	34.8	3922.9	47.7	385.3	4358.2	13,180.1

## Data Availability

The original contributions presented in this study are included in the article material. Further inquiries can be directed at the corresponding author(s).
